# Motivational adaptation in English grammar learning: a mixed-methods study of students during the transition to senior high school

**DOI:** 10.3389/fpsyg.2025.1703576

**Published:** 2026-01-07

**Authors:** Zhuojun Zhuang, Di Wang

**Affiliations:** School of Education, Shanghai International Studies University, Shanghai, China

**Keywords:** English grammar learning, expectancy-value theory, mixed-methods study, motivational adaptation, senior high school transition

## Abstract

The transition to senior high school represents a critical developmental period where adolescents face increased academic demands that can challenge their psychological motivation. Given the important role of motivation in language learning, this study examines how students adjust their learning motivation during the transition. Drawing on expectancy-value theory (EVT), the mixed-methods study aims to explore senior high school freshmen’s motivational adaptation in English grammar learning during the transition and the factors influencing the process. Pre- and post-semester questionnaires and follow-up interviews were used to examine changes in students’ motivational beliefs and to identify influential factors. Quantitative results showed significant increases in intrinsic value, utility value, and expectancies for success, while attainment value and perceived task difficulty remained stable. Qualitative analysis identified four key influences: students’ affective attitudes, perceived increase in grammar difficulty, targeted instructional support, and perceived self-worth. This study contributes to the understanding of motivational adaptation during high-pressure academic transitions and highlights that well-designed grammar instruction plays a key role in facilitating smoother psychological adjustment during school transitions.

## 1 Introduction

The transition to a new school is an influential developmental event during adolescence (Barber and Olsen, 2004). This period is often accompanied by substantial changes and challenges (Hirsch and DuBois, 1992) that can impact students’ psychological wellbeing and academic achievements. Specifically, the transition from junior to senior high school is frequently associated with declines in academic performance (Barone et al., 1991; Felner et al., 1981; Isakson and Jarvis, 1999), and socioemotional functioning, including increased feelings of depression and anxiety (Benner and Graham, 2009; Benner et al., 2017; Liu et al., 2023; Newman et al., 2007). The success of this adaptation also has long-term consequences (Garner and Moots, 2018), influencing school drop-out rates (Alspaugh, 1998), and students’ future educational opportunities (Mac Iver and Messel, 2013). Given the immediate and profound effects that transitioning has on students, it is essential to explore the motivational processes that support students’ adaptation during this critical period.

In the context of English education in China, the transition from junior to senior high school demands a sudden and significant increase in the complexity of grammar content, coupled with higher expectations for language proficiency (Li, 2025). This abrupt shift in the academic environment can create a mismatch with students’ previously held competence beliefs and learning strategies, potentially disrupting their motivation and engagement. Currently, numerous difficulties in learning English grammar among Chinese learners have been identified (e.g., Li et al., 2024; Nicoladis et al., 2020). For example, Li et al. (2024) found that the structural differences between English and Chinese increase the difficulty in tense learning, as Chinese is a tenseless language. However, the motivational challenges and psychological adaptations students face when learning English grammar during the transition to senior high school have received far less attention.

To address this gap, this mixed-methods study aims to employ expectancy-value theory (EVT) (Eccles and Wigfield, 2002) to investigate how 1st-year senior high school students adapt motivationally to English grammar learning and what factors impact their adaptation process. According to EVT, students’ expectancies for success and subjective task values are key predictors of learning intentions and academic achievements (Wigfield and Eccles, 2000). As these beliefs change in response to new academic demands, they reflect how students adjust motivationally to their learning context. In this study, positive motivational change is considered the indicator of successful academic adaptation.

This investigation aims to enrich our understanding of motivational adaptation in second language contexts, with a particular focus on grammar learning during a key educational transition.

Specifically, the research questions of the study are:

*RQ1*: How do students’ expectancy-value-related perceptions of English grammar learning change after a semester of transition?

*RQ2*: What are the key factors influencing students’ adaptation to English grammar learning during the transition to senior high school?

## 2 Literature review

### 2.1 Negative effects of educational transitions on students

Ellis and Del Giudice (2019) found that adapting to a new environment or role often posed challenges for individuals. Akos and Galassi (2004) identified three key challenges that students met with during the transition from junior to senior high school: academic, procedural, and social challenges, all of which hindered the process of transition.

Academically, students struggled with more homework and tougher classes (Akos and Galassi, 2004). The high school transition was mostly investigated through the perspective of students’ academic performance (Benner, 2011). Numerous studies found that students’ academic achievement experienced a drop during transition (see, e.g., Benner and Graham, 2009; Felner et al., 1981; Isakson and Jarvis, 1999; Suldo et al., 2019). For instance, Felner et al. (1981) observed that the normative transition to senior high school was negatively associated with students’ academic performance and attendance. These findings were consistent with those of Benner and Graham’s (2009), who reported that although students expressed a greater preference for senior high school compared to junior high immediately after entering the new school, this initial enthusiasm failed to prevent students from decreased grades and increased absences. The pattern was further supported by Isakson and Jarvis’s (1999) short-term longitudinal study, which indicated a decline in students’ Grade Point Average (GPA) during the transition.

Procedural challenges focused on the adaptation to the organizational structure and physical layout of a new school (Akos and Galassi, 2004). In their study of 320 ninth-grade students in the United States, Akos and Galassi (2004) found that 59% reported getting lost as one of their primary concerns during the transition. Similarly, Weiss (2001) noted that turbulence resulting from school disorganization, such as textbook shortages and schedule changes beyond students’ control, brought about students’ adjustment difficulty. Additionally, Barber and Olsen (2004) reported that the transition to senior high school led to a decline in students’ perceived quality of the school environment. Specifically, a perceived decrease in teacher support was significantly associated with higher levels of student depression.

Socially, students had to adapt to new peer environments and deal with the risks of conflict (Akos and Galassi, 2004). Faced with a new environment, senior high school freshmen reported experiencing a range of negative emotions, such as increased levels of loneliness, anxiety, and depression (Barber and Olsen, 2004; Benner and Graham, 2009; Benner et al., 2017). Benner et al. (2017) specifically found that boys were more vulnerable to depressive symptoms, which might have been attributed to the limited attention given to boys regarding the mental health challenges they faced. Additionally, the transitional period also involved a process of social repositioning, in which students reassessed and renegotiated their identity and place within a new peer and institutional context. Some senior high school freshmen were found to experience a decline in their subjective social status during their first semester, indicating a perceived drop in their social standing within the peer group (Liu et al., 2023). However, Kinney (1993) found that previously less popular students experienced positive changes after the transition to high school because of a more mixed social structure. They were more confident and were less worried about if they were popular or not. To address the challenges students faced during this transitional period, Benner and her colleagues (Benner et al., 2017) identified that both friend support and school belonging were positively related with students’ socioemotional functioning.

### 2.2 Challenges in English grammar instruction and transition in China

The arguments and debates surrounding grammar teaching suggested that TESOL methodologists struggled to offer consistent suggestions to teachers about the role of grammar in language teaching over the past 25 years (Celce-Murcia, 1991). In China, Cheng (2020) emphasized that both grammar teaching and learning were hard, and the effects of grammar teaching were far from satisfactory. Hence, it was not unexpected that a lot of problems occurred during the process of English grammar instruction.

Cheng (2020), based on practical classroom teaching cases, identified several problems that frontline teachers were faced with: the inappropriate setting of context, the lack of authenticity of language materials, the rigid instruction of grammar rules, and the undue emphasis on emphatic sentences. Song and Dong (2015) also posed additional problems like students’ poor autonomy, formalistic cooperation with each other, and inefficient classroom progress. Besides, teachers’ understanding of grammar also needed improvement (Lin and Jia, 2015). Many teachers relied primarily on traditional grammar knowledge, ignoring the advancements in linguistics and language teaching theories that extended the understanding of English grammar concepts.

While grammar instruction faced classroom-level challenges, these were further complicated by broader transitional difficulty in English education between junior and senior high school. In a large-scale national survey of 392 senior high school English teachers across 26 provinces in China, Chang et al. (2021) found that 55.87% of the respondents believed there were notable problems in the transition between junior and senior high school English curricula. The issues were primarily due to significant differences in vocabulary load, instructional methods, and curriculum difficulty between the two stages. Additionally, in interviews with six English education experts, three identified the difficult transition between junior and senior high school as a major concern in China’s English education system. Wu’s (2017) argument further echoed these findings, suggesting that the tough transition was a key contributor to students’ declining academic performance.

Given that grammar was heavily emphasized in senior high school English instruction due to exam pressure (Chang et al., 2021), any discontinuity in curriculum during the transition became particularly disruptive.

### 2.3 Expectancy-value theory

#### 2.3.1 Overview of EVT

The expectancy-value theory (EVT) proposed by Eccles (1983) was developed from Atkinson’s (1957) expectancy-value model, retaining its core elements while expanding to include broader psychological and social dimensions (Eccles and Wigfield, 2002). This study focuses on three major constructs from the model: task values, expectancies for success, and perceived task difficulty.

Four different components consist of task values: attainment value, intrinsic interest value, extrinsic utility value, and cost (Eccles, 1983). Attainment value is defined as the importance of doing well on one task. Intrinsic value can be considered as the pleasure one gets from doing the task. Utility value refers to the usefulness of a task, like how a task benefits an individual’s future plans. Cost means how the decision to engage in one activity (e.g., homework) limits access to other activities (e.g., playing computer games), evaluations of the efforts required to finish the task, and emotional cost. Most of the empirical work paid attention to the first three constructs (Eccles and Wigfield, 2002), so we limit our discussion to them.

In addition to task values, expectancies for success are another key component of EVT. Eccles (1983) defined this construct as children’s beliefs about how well they will do on upcoming tasks, whether in the near future or in the long term. Additionally, perceived task difficulty, a part of the task-specific beliefs, can be described as students’ evaluation of how hard a certain task is. Beliefs about tasks can influence expectancies and values (Eccles, 1983). Hence, it is included in the present study given its significance in the model.

#### 2.3.2 Applying EVT to grammar learning motivation

EVT was widely applied to investigate changes in students’ motivation during the educational transitions (see, e.g., Bargmann et al., 2022; Lazarides et al., 2022; Mayerhofer et al., 2024; Wigfield et al., 1991). For instance, Wigfield et al. (1991) examined how young adolescents’ self-concepts of ability and liking for English, two key constructs in EVT, changed during the transition to junior high school. Applying a four-wave design with data collected twice before and twice after the transition, the research found that both constructs declined across the transition. Specifically, students’ English-ability perceptions were significantly lower in junior high school than in primary school. Additionally, although students’ liking for English increased in the second semester of junior high school, it remained below the level observed in the final semester of primary school. These findings showed EVT’s sensitivity to motivational changes resulting from structural shifts in schooling.

While the use of EVT in second language acquisition research was not widely explored, the existing studies yielded some important results (Dong et al., 2022). EVT was found to be effective in explaining why and how learners were motivated to study a second language (Nagle, 2021) and was employed in language learning studies (see, e.g., Arens et al., 2019; Mori and Gobel, 2006; Nagle, 2021). For instance, Nagle (2021) employed EVT to investigate university students’ L2 learning motivation during a critical transition point in a language curriculum. The findings showed that the key constructs of EVT had significant associations with students’ language learning motivation, persistence, and achievement.

In the Chinese senior high school context, as discussed above, 1st-year students often struggle with grammar because of curriculum discontinuity and the increased complexity of grammatical concepts. EVT suggests that expectancies and task values are influenced by perceived task difficulty (Wigfield and Eccles, 2000). The increased complexity of grammar concepts in senior high school is likely to increase learning difficulty, and ultimately lower students’ expectancies and perceived task values. Besides, Fischoff et al. (1982) suggested that students attached higher task values to activities with which they were familiar. Hence, curriculum discontinuity may reduce their familiarity with senior high school grammar tasks, lowering their perceived task values. From this perspective, structural changes in grammar instruction in senior high school can influence students’ expectancies for success and task values. As these beliefs change, their motivation increases or decreases accordingly.

Given EVT’s focus on learners’ expectancies for success and on multi-dimensional values attached to learning tasks (Wang and Xue, 2022; Wigfield and Eccles, 2000), and its successful application in research on educational transitions and language learning, EVT provides an appropriate framework for examining motivational adaptation in grammar learning. By employing this theoretical framework, the study identifies key motivational challenges that 1st-year senior high school students face. This can inform educators’ development of targeted instructional strategies, which can help students better adapt to senior high school English grammar learning.

## 3 Methodology

### 3.1 Mixed-methods study design

The mixed-methods study was conducted from September 2024 to January 2025. It consisted of two stages. The quantitative stage aimed to examine changes in students’ motivation toward English grammar learning. Questionnaires were administered to assess five dimensions of EVT. Based on the quantitative findings, the qualitative stage involved semi-structured interviews to explore the factors influencing students’ transitions and their subjective evaluations of their grammar learning during the transition to senior high school.

### 3.2 Contexts

The study was conducted in a public senior high school located in an urban area in Shanghai, an international metropolis in eastern China. During the semester in which the study was conducted, participants at the school had six English classes every week, each lasting 40 min. To make sense of the specific instructional practices observed in this study, it is necessary to first understand the broader educational context.

Given the city’s global status, English is highly prioritized in Shanghai, and students start learning English from Grade 1 in primary school. Across the country, English is a compulsory subject in both senior high school and college entrance examinations. To standardize English instruction nationwide, the Ministry of Education has published two sets of English curriculum standards, one for compulsory education (primary and junior high school) (Ministry of Education of the People’s Republic of China, 2022) and the other for senior high school (Ministry of Education of the People’s Republic of China, 2020).

Both sets of curriculum standards emphasize the activity-based approach to English learning, which highlights that English instruction should be organized around meaning-oriented and discourse-based learning activities. This approach was developed by Chinese scholars to guide Chinese English instruction and introduced a three-level progression of activities, namely, learning and understanding (e.g., Students read an email and identify five instances of the present continuous used to express future arrangements), applying and practicing (e.g., In groups, students complete the missing parts of a “weekend plan” by adding three sentences in the present continuous), and transferring and creating (e.g., Students design an “Unexpected Shanghai” one-day tour. They write six original invitation sentences using the present continuous and record a 60-s English vlog). This structured sequence enables students to gradually develop basic grammatical concepts, learn to use different grammatical forms across contexts, and ultimately have the ability to express their ideas flexibly.

In Shanghai, the college entrance examination assesses grammatical ability through discourse. This is in line with the curriculum standards’ emphasis on contextualized and meaningful use of grammar, which reduces the tension between curriculum goals and assessment demands, creating more space for discourse-oriented grammar teaching.

However, the senior high school English entrance examination in Shanghai (commonly known as *Zhongkao*, the city-wide standardized examination taken at the end of junior secondary schooling for admission into senior high school) assesses grammar through multiple-choice items, which lack sufficient context. This format provides limited opportunities to evaluate students’ contextualized use of grammar, bringing about extensive mechanical drilling in practice. Therefore, it raises challenges to the implementation of the activity-based approach in junior high school classrooms.

Within the broader context, English instruction in the participating school closely followed the prescribed textbook. Each unit began with a reading text, followed by the grammar section, which introduced one specific grammatical structure. In this section, explicit grammatical rules were presented alongside contextualized examples to help students understand and apply the target structure effectively. Grammar instruction proceeded strictly according to the sequence of the textbook, with no content being taught in advance or omitted. The activity-based approach to English learning was also implemented in daily instruction, with teachers designing real-life contexts and learning activities to help students develop their grammatical competence gradually.

### 3.3 Participants

Applying a combination of purposive and convenience sampling, 80 students (40 boys and 40 girls) from a key senior high school were invited to complete the questionnaires. They had all completed their compulsory education in local junior high schools in Shanghai and took the city’s senior high school entrance examination, 2 months before entering senior high school. All participants were 1st-year students in their first semester of senior high school. They were from two parallel classes and taught by the same English teacher.

The participants’ baseline English proficiency was assessed through their English scores on the senior high school entrance examination, with a mean score of 138.4 out of 150 (see Table 1). Based on the score and the learning objectives introduced in the *Compulsory Education’s English Curriculum Standards (2022 Edition)* (Ministry of Education of the People’s Republic of China, 2022), the participants’ English proficiency can be estimated to correspond to Level B1 of the Common European Framework of Reference for Languages (Council of Europe, 2020). While all students had a basic foundation in English, little was known about how they adapted to the increased grammatical demands of senior high school.

**Table 1:** Baseline English proficiency of the participants (*N* = 80).

Measure	Mean	SD	Max	Min
Senior high school entrance exam English score (out of 150)	138.40	5.72	147.00	120.00

Estimated CEFR level of the participants: B1.

During the qualitative phase, maximum variation sampling (Patton, 2014), a purposive sampling method, was adopted to ensure diversity in students’ academic performance. Five participants (two boys and three girls) who had completed the questionnaires were invited to represent different achievement levels, allowing for a wider understanding of how students with various academic levels perceived their motivational changes in grammar learning (see Table 2). Additionally, an experienced teacher with nearly 30 years of teaching experience at the same school was invited to participate in the interview. Given the critical role teachers play in school environment and their responsibility in supporting students’ adaptation to senior high school learning, the teacher’s perspective is equally important (Iacobescu et al., 2024).

**Table 2:** Basic information of the five interviewees.

Pseudonym	Gender	Background characteristics
Allen	Male	Lower-achieving
Anna	Female	High-achieving
Rachel	Female	Fluctuating
Bob	Male	Average
Jasmine	Female	High-achieving
Helen (teacher)	Female	Nearly 30 years of teaching experience

### 3.4 Procedure

The research was conducted over a 4-month period, starting from the beginning to the end of the semester. This study was approved by the Institutional Review Board of Shanghai International Studies University (approval number: SISUGJ20251009). During the quantitative phase, the questionnaire was administered twice, once at the beginning of the semester and the other at the end. The qualitative phase started 5 days after the administration of the second questionnaire. Semi-structured interviews were conducted with five students and one experienced teacher. The following subsections describe the two stages in detail.

#### 3.4.1 Quantitative phase

To investigate students’ changes in grammar learning motivation, the same questionnaire was administered twice, at the beginning and the end of the semester, respectively. The full questionnaire is provided in Appendix A. Specifically, participants completed the first questionnaire 2 weeks after they began senior high school, and the second one 3 days after their final exams. Both questionnaires were distributed through an online survey platform Wenjuanxing. Before completing the survey, participants were informed of the study’s purpose, procedures, and confidentiality measures, and all voluntarily agreed to participate. The questionnaire was originally developed and administered in Chinese to ensure comprehensibility for senior high school participants.

The questionnaire adopted a four-point Likert Scale, ranging from (1) “strongly disagree” to (4) “strongly agree.” Consisting of five sub-scales, the questionnaire focused on the key constructs within EVT (Eccles and Wigfield, 2002), namely, intrinsic interest value (sample item: I think senior high school English grammar learning is enjoyable), attainment value (sample item: Being good at senior high school English grammar is important to me), extrinsic utility value (sample item: English is very helpful in my daily life), expectancies for success (sample item: I believe I can successfully learn new English grammar topics), and perceived task difficulty (sample item: I find English grammar difficult to learn).

A four-point Likert scale was chosen for two reasons. First, during the pre-test, students reported difficulty judging the subtle differences between six options. Besides, given the middle response style among Chinese students, they are more likely to choose neutral or middle responses when completing questionnaires (Chen et al., 1995; Lozano et al., 2008). Therefore, a four-point Likert scale was employed to reduce the participants’ cognitive load and to avoid providing middle options.

To ensure the quality of the questionnaire, a pre-test was conducted with five students (two boys and three girls) to examine the clarity and comprehensibility of the questionnaire items. They were from the same school as the main study but did not participate in the main data collection. Their English scores on the senior high school entrance examination were comparable to the mean score of the main study participants (within 1–2 points). They were invited to complete the draft questionnaire and provide feedback on the wording, clarity, and length of each item. Minor revisions were then made to improve the wording of the final version. For instance, several expressions were slightly adjusted to be more natural and comprehensible in the Chinese context (e.g., “invest a lot of efforts” was changed to “put in a lot of efforts”). Besides, two domain experts in English language teaching reviewed the questionnaire to ensure content validity. Their suggestions were accepted to improve the relevance of the items and the overall instrument quality. For example, one expert advised making the item more specific to grammar learning, so the phrase “especially in terms of grammar” was added to “I am confident in learning senior high school English.” The final version of the questionnaire, administered to 80 students in the study, demonstrated good internal consistency (Cronbach’s *α* = 0.92).

#### 3.4.2 Qualitative phase

Face-to-face semi-structured interviews were conducted to explore the factors influencing students’ motivational adaptation. The interviews were conducted in a quiet classroom on campus to provide a familiar and comfortable setting for the interviewees.

For the students’ interviews, the focus was primarily on their perceptions of senior high school English grammar learning, guided by key constructs of EVT (sample question: Concerning the transition between junior and senior high school English grammar, do you feel you have encountered any difficulties? Why or why not? Have you overcome these difficulties?). The teacher interview focused on the common challenges students face during their transition to senior high school, the causes of these challenges, and the corresponding strategies used to deal with these issues through curriculum design and instructional methods. The teacher was also asked to share her insights on the differences between grammar teaching and learning at the junior and senior high school levels (sample question: In your grammar teaching, especially during the transition from junior high to senior high, have you encountered any common difficulties among students?). The interviews lasted between 39 and 50 min, with an average of 45 min. For the teacher and student interview guides (see Appendices B, C).

The interviews were designed and conducted in Mandarin. With their consent, all interviews were audio-recorded and transcribed verbatim. Participants were informed about the purpose of the interviews and assured of their anonymity throughout the process.

Both the quantitative and qualitative phases were carried out with the consent of the participants’ class teachers. All responses were subsequently translated into English by the first author.

### 3.5 Data analysis

As the qualitative data were not normally distributed, the Wilcoxon signed-rank test was applied to assess changes in students’ adaptation to English grammar learning from the beginning to the end of the first semester. Each pair of scores (Questionnaire 1 and 2) was collected from a single student, and different pairs were treated as independent observations. No outliers that required exclusion were found. Results were presented as median and interquartile range (IQR). All tests were two-sided and a *p* < 0.05 was considered to be statistically significant. The analysis was performed using IBM SPSS Statistics version 29.0 (IBM Corp., Armonk, NY, USA).

Thematic analysis (Braun and Clarke, 2021) was used to identify the key contributing factors to students’ adaptation to English grammar during the transition period. Following the six-phase approach, an inductive coding strategy was employed to allow themes to emerge from the data. The data were coded manually. To ensure coding reliability, two researchers independently conducted open coding on 50% of the transcripts (*n* = 3) and calculated coding consistency using Krippendorff’s alpha (*α* = 0.83). Discrepancies were discussed and solved through discussions between the researchers. Then, the first author coded the remaining transcripts. Themes were generated through code aggregation and constant comparison. The corresponding author rigorously checked the themes and the final version was confirmed through joint review. A detailed codebook, including themes, codes, definitions, and quotes, is provided in Appendix D. The preliminary analyses of findings were also returned to all the interviewees for member checking. They were invited to confirm whether the interpretations accurately reflect their feelings and thoughts. Feedback was collected through WeChat. All participants confirmed the credibility of the interpretations, and no revisions were proposed.

## 4 Results

### 4.1 Adaptation to expectancy–value motivation in English grammar learning during the transition

To address Research Question 1, this section presents the changes in students’ expectancy–value perceptions of English grammar learning over the semester.

#### 4.1.1 Comparative analyses of questionnaire one and two

Tables 3, 4 exhibited the descriptive statistics of students’ motivational changes in English grammar learning at the beginning (T1) and the end (T2) of their first semester in high school. Overall, the mean scores of most scales witnessed an increase from T1 to T2. For instance, intrinsic interest value increased from M = 2.45 (SD = 0.84) to M = 3.13 (SD = 0.60), and extrinsic utility value rose from M = 2.79 (SD = 0.80) to M = 3.12 (SD = 0.55). Similarly, expectancies for success increased from M = 2.45 (SD = 0.83) to M = 3.06 (SD = 0.85).

**Table 3:** The results of the descriptive analysis at Time 1 (T1).

Dimension	Max	Min	Mean	SD
Intrinsic interest value	4.00	1.00	2.45	0.84
Attainment value	4.00	1.00	3.22	0.82
Extrinsic utility value	4.00	1.00	2.79	0.80
Expectancies for success	4.00	1.00	2.45	0.83
Perceived task difficulty	4.00	1.00	3.37	0.64

**Table 4:** The results of the descriptive analysis at Time 2 (T2).

Dimension	Max	Min	Mean	SD
Intrinsic interest value	4.00	1.00	3.13	0.60
Attainment value	4.00	1.00	3.27	0.62
Extrinsic utility value	4.00	1.00	3.12	0.55
Expectancies for success	4.00	1.00	3.06	0.85
Perceived task difficulty	4.00	1.00	3.21	0.62

In contrast, attainment value remained relatively stable across the two time points (M = 3.22, SD = 0.82 at T1; M = 3.27, SD = 0.62 at T2). Perceived task difficulty also showed little change (M = 3.37, SD = 0.64 at T1; M = 3.21, SD = 0.62 at T2).

These results suggested that students increasingly perceived grammar learning as engaging, recognized its practical benefits, and developed greater confidence in their ability to master it. In contrast, their perceived importance of achievement and their perceptions of task difficulty remained relatively stable.

The results from the Wilcoxon signed-rank test (see Table 5) showed there was a statistically significant increase in intrinsic interest value from the beginning of the semester, with the median (IQR) rising from 2.43 (1.86–3.14) to 3.29 (2.86–3.57), *p* < 0.001, *r* = 0.56, which constituted a large effect in educational psychology. These findings indicated that the participants were more engaged and interested in English grammar learning and that the increase in this dimension during the semester held practical educational significance.

**Table 5:** Comparison of questionnaire scores at the beginning and end of the semester.

Dimension	Questionnaire 1 Median (*N* = 80)	(IQR)	Questionnaire 2 Median (*N* = 80)	(IQR)	*Z*	p*	*r*
Intrinsic interest value	2.43	(1.86, 3.14)	3.29	(2.86, 3.57)	5.02	<0.001	0.56
Attainment value	3.33	(2.67, 4.00)	3.33	(3.00, 3.67)	0.35	0.730	0.04
Extrinsic utility value	2.75	(2.25, 3.50)	3.25	(2.75, 3.50)	2.71	0.007	0.30
Expectancies for success	2.50	(2.00, 3.00)	3.00	(2.50, 3.50)	3.74	<0.001	0.42
Perceived task difficulty	3.50	(3.00, 4.00)	3.50	(3.00, 3.50)	−1.88	0.061	0.21

*From Wilcoxon signed-rank test.

A significant increase was also observed in extrinsic utility value, rising from a median of 2.75 (IQR = 2.25–3.50) at the beginning to 3.25 (IQR = 2.75–3.50) at the end of the semester, *p* = 0.007, *r* = 0.30, indicating a medium effect size. The results suggested a moderate and practically meaningful increase in students’ perception of the usefulness of English grammar learning.

Furthermore, students’ expectancies for success also witnessed a significant increase from the beginning of the semester to the end of the semester, with the median (IQR) rising from 2.50 (2.00–3.00) to 3.00 (2.50–3.50), *p* < 0.001, *r* = 0.42, signaling a medium effect size. The findings revealed that students were more confident that they could do well in English grammar over a semester of learning.

In contrast, it was found that from the beginning of the semester to the end of the semester, the perceived attainment value did not increase significantly. The median (IQR) was 3.33 (2.67–4.00) at the beginning and 3.33 (3.00–3.67) at the end of the semester, *p* = 0.730, *r* = 0.04, showing a rather small effect size. These suggested that students’ perceptions of the importance of English grammar learning remained relatively stable over the course of a transitional semester.

Regarding the perceived task difficulty, the median remained the same from the beginning to the end of the semester (3.50), while the IQR narrowed from (3.00–4.00) to (3.00–3.50), *p* = 0.061, *r* = 0.21, indicating a small effect size. The results reflected that students’ perceptions of the difficulty of grammar learning remained largely unchanged over the semester.

#### 4.1.2 The interviewees’ responses

The interview data exhibited varied adaptation experiences. Among the five interviewees, Allen and Anna reported that they had not fully adjusted to senior high school English grammar learning. While Allen struggled with English due to a weaker academic foundation, Anna, despite being highly proficient and consistently achieving top English scores, also felt that she had not fully adjusted. This contrast suggested that students with lower academic achievement may require more time and support to adapt, and high-achieving students would still experience psychological barriers during transition. Anna’s case particularly highlighted the disconnection that can exist between objective academic performance and students’ subjective learning perception. The remaining three interviewees, Rachel, Bob, and Jasmine, indicated that they had generally adjusted to the English grammar learning in senior high school:

*I feel like I have a deeper understanding in attributive clause. Whenever I manage to solve a tough grammatical problem, I feel really proud of myself (Excerpt from the interview with Bob).*

Allen also shared his development in the grasp of grammar knowledge:

*I have a better understanding of sentence structure as well as the application of relative clauses, adverbial clauses, and object clauses (Excerpt from the interview with Allen).*

### 4.2 The influential factors behind the transition of English grammar learning to senior high school

To address Research Question 2, this section identifies the factors influencing students’ adaptation to expectancy-value motivation during the transition.

#### 4.2.1 Affective attitudes towards English grammar learning

According to the interviewees, students with a more positive attitude towards high school English grammar learning tended to experience a more successful transition. This theme corresponds to the intrinsic interest value component in EVT (Eccles and Wigfield, 2002). Table 6 shows the qualitative codes related to students’ affective responses to grammar learning.

**Table 6:** The themes, codes, definitions and representative quotations concerning students’ affective attitudes towards English grammar learning.

Theme	Code	Definition	Quote
Affective attitudes towards English grammar learning	Dislike and avoidance of English learning	Strong negative emotions toward English learning and a clear tendency to avoid it	“I do not like English. To be honest, learning English is something I do not really want to do. I would rather practice two hours on math than memorize English words for half an hour.”
	Emotional adjustment toward acceptance	The attempt to transform resistance into a more accepting attitude toward English learning	“But I am trying to turn English learning into something acceptable for me.”
	Enthusiasm toward grammar learning	Strong motivation to master grammar despite its challenges	“I laid much more emphasis on English grammar learning. Although it is challenging, I am making every effort to be the ‘grammar master’.”

Allen, who self-reported difficulty in adjusting to grammar learning, expressed emotional avoidance in his attitude toward the subject.:

*I do not like English. To be honest, learning English is something I do not really want to do. I would rather practice two hours on math than memorize English words for half an hour. But I am trying to turn English learning into something acceptable for me (Excerpt from the interview with Allen).*

Similarly, Bob, who had an intermediate level of English proficiency, expressed an average attitude towards English learning, reflecting a moderate level of intrinsic interest.

In contrast, students who excelled in English showed more positive affective attitudes towards their grammar learning. Jasmine shared her positive affective engagement:

*I laid much more emphasis on English grammar learning. Although it is challenging, I am making every effort to be the “grammar master.” (Excerpt from the interview with Jasmine).*

It was not uneasy to find that students’ positive attitudes toward English grammar learning could influence their learning outcomes. A likely explanation is that such attitudes encourage greater effort and dedication, helping students adapt more smoothly during the transition period.

#### 4.2.2 Perceived increase in grammar difficulty

All interviewees reported a sharp contrast between grammar instruction in junior and senior high school, indicating a significant perceived increase in grammar learning difficulty. This theme reflects the perceived task difficulty component in EVT (Eccles, 1983).

In junior high school, grammar was often learned through repetitive drills without explicit conceptual instruction. However, in senior high school, grammar learning became more structured and concept-driven, which required a higher level of abstract reasoning and mental effort (see Table 7). For instance, Allen shared his experience:

**Table 7:** The themes, codes, definitions and representative quotations concerning perceived increase in grammar difficulty.

Theme	Code	Definition	Quote
Perceived increase in grammar difficulty	Increased task complexity and learning demands	Greater analytical and integrative ability demands in senior high school grammar learning	“I learned English grammar mainly by doing exercises in junior high school. Although there was no systematic instruction, I still felt it was simple and I scored high marks. In senior high school, grammar is really important. Without a good command of grammar, it is hard to perform well in reading tasks.”
	From drilling to conceptual understanding	A transition from mechanical drills to concept-based grammar learning	“My English teacher taught me grammar mainly by drilling in junior high school, without much focus on explaining the concepts behind it. Therefore, I just kept practicing different exercises. In senior high school the grammar concepts are clearly explained in class. Now, I think understanding the concepts and using them correctly in different contexts are more important.”
	Lack of conceptual preparation in junior high school	Increased senior high grammar learning difficulty caused by inadequate junior high grammar preparation	“One common problem most students encounter in senior high school is the lack of conceptual knowledge of English grammar. We need to take a lot of efforts to help them first understand these concepts. This step should have been done when they were in junior high school.”
	Perceived significant increase in difficulty	The perceived increase in English learning difficulty in senior high school	“In junior high school, English was really easy. I was quite satisfied with my senior high school entrance examination results. But in senior high school, it’s a different story. The difficulty level has risen so much.”

*In junior high school, English was really easy. I was quite satisfied with my senior high school entrance examination results. But in senior high school, it’s a different story. The difficulty level has risen so much (Excerpt from the interview with Allen).*

Bob also emphasized the ease of grammar learning in junior high school, remarking that he did not find any grammar points particularly challenging at that stage.

Anna’s change in her view of grammar learning also reflected the perceived increase in learning difficulty. She shifted from treating grammar as simple to considering it as a prerequisite for doing well in reading tasks. This reappraisal of grammar as a useful tool for successful reading indicates an increased emphasis on extrinsic utility value (Eccles and Wigfield, 2002).

*I learned English grammar mainly by doing exercises in junior high school. Although there was no systematic instruction, I still felt it was simple and I scored high marks. In senior high school, grammar is really important. Without a good command of grammar, it is hard to perform well in reading tasks (Excerpt from the interview with Anna).*

Their words indicated an objective increase in the difficulty of senior high school grammar. However, students felt this increase more strongly due to their insufficient preparation in junior high school, making the transition particularly tough. Helen considered this a common challenge students faced in adapting to senior high school grammar instruction:

*One common problem most students encounter in senior high school is the lack of conceptual knowledge of English grammar. We need to take a lot of effort to help them first understand these concepts. This step should have been done when they were in junior high school (Excerpt from the interview with Helen).*

According to Helen, many students entered senior high school without a solid conceptual foundation, which hindered their ability to deal with the more complex grammar curriculum.

The huge changes in grammar instruction methods also intensified students’ perceptions of the increased learning difficulty. One key change was the increased emphasis on textbooks as central tools for grammar instruction in senior high school. Helen, the teacher interviewed, emphasized the importance of following the textbook structure to help students contextualize grammar within reading materials. Compared to previous strategies that emphasized repetition, the new approach demanded greater cognitive engagement and a higher ability to apply grammar in context, which in turn led to changes in students’ learning methods.

Rachel described how this change altered her learning strategy:

*My English teacher taught me grammar mainly by drilling in junior high school, without much focus on explaining the concepts behind it. Therefore, I just kept practicing different exercises. In senior high school the grammar concepts are clearly explained in class. Now, I think understanding the concepts and using them correctly in different contexts is more important (Excerpt from the interview with Rachel).*

The insufficient conceptual preparation in junior high school and the significant changes in the teaching and learning of grammar in senior high school made the objectively more demanding grammar curriculum feel even more difficult for students. These factors complicated students’ adjustment and raised challenges during the transition.

#### 4.2.3 Instructional support from teachers

Both student and teacher interviewees emphasized that effective instructional support played a critical role in helping learners adapt to senior high school grammar learning (see Table 8).

**Table 8:** The themes, codes, definitions and representative quotations concerning instructional support from teachers.

Theme	Code	Definition	Quote
Instructional support from teachers	Peer learning through example display	The use of students’ work as examples to facilitate grammar learning	“My teacher displays my classmates’ homework in the form of photos, allowing us to learn from their problem-solving methods, since there are marks on the papers.”
	Detailed conceptual explanation	Support for grammar learning by detailed explanations of grammatical concepts	“She also goes through each question in detail, helping us understand the underlying grammatical concepts.”
	Active student participation in error analysis	Active explanation of grammatical knowledge to peers	“One effective method my English teacher uses is called the ‘Error Case Report.’ Each student selects a representative grammar error from their daily practice, prepares relevant materials, and explains it to the whole class.”
	Learning benefits of reflective error analysis	Benefits of reflective error analysis for students’ understanding of grammar rules	“This process not only deepens my understanding of the error itself but also helps me recognize similar issues in others’ work.”

Helen, the teacher participant, emphasized the importance of concept-focused instruction and individualized scaffolding. In her view, teaching should go beyond rote memorization and actively guide students to analyze sentence structures and internalize grammar rules. Students’ reflections echoed this view. Bob explained how his teacher’s use of visual scaffolding and detailed analysis improved his conceptual understanding:

*My teacher displays my classmates’ homework in the form of photos, allowing us to learn from their problem-solving methods, since there are marks on the papers. She also goes through each question in detail, helping us understand the underlying grammatical concepts (Excerpt from the interview with Bob).*

Similarly, Anna described a classroom practice that not only reinforced her understanding but also fostered metacognitive awareness:

*One effective method my English teacher uses is called the “Error Case Report.” Each student selects a representative grammar error from their daily practice, prepares relevant materials, and explains it to the whole class. This process not only deepens my understanding of the error itself but also helps me recognize similar issues in others’ work (Excerpt from the interview with Anna).*

Therefore, effective guidance from teachers was important for supporting a smoother transition, as it enabled students to develop confidence in grammar learning by making it feel less difficult, thus increasing their expectancies for success (Eccles and Wigfield, 2002).

#### 4.2.4 Perceived self-worth value

As shown in Table 9, perceived self-worth emerged as a motivational factor influencing students’ adaptation to English grammar learning. This theme reflects the attainment value component in EVT (Eccles and Wigfield, 2002), in which students are motivated by the importance of doing well for personal pride. According to the interview, students who closely associated academic success with their personal or social image were more likely to invest more effort in order to avoid failure and maintain self-esteem. Allen shared this connection directly:

**Table 9:** The themes, codes, definitions and representative quotations concerning perceived self-worth value.

Theme	Code	Definition	Quote
Perceived self-worth value	Effort motivated by self-worth concerns	Study motivation related to maintaining a positive peer image	“I am the class monitor, and if I perform poorly in English, then it will be really embarrassing. Therefore, I need to work harder to catch up with the other classmates.”

*I am the class monitor, and if I perform poorly in English, then it will be really embarrassing. Therefore, I need to work harder to catch up with the other classmates (Excerpt from the first interview with Allen).*

Allen’s comment reflected a strong internal pressure to maintain a positive academic image, especially in front of peers. If a student had a higher perceived self-worth, then he or she would be more likely to put in extra effort in order not to “lose face.” This drive could positively impact the transition process by motivating the students to work harder and overcome challenges.

## 5 Discussion

The purpose of the paper is to explore students’ adaptation to English grammar learning in senior high school during the transitional period, as well as the key factors influencing this process. The findings reveal several key trends. Students’ perceived intrinsic interest value, extrinsic utility value, and expectancies for success all increased by the end of the semester, suggesting improvements in engagement and confidence. Unexpectedly, attainment value and perceived task difficulty did not exhibit significant changes, indicating that students’ beliefs in these areas may take longer to develop. In addition, qualitative data identified four major factors that influenced students’ adaptation during transition: students’ affective attitudes towards English grammar learning, perceived increase in grammar difficulty, instructional support from teachers, and perceived self-worth value.

The study found a significant increase in students’ intrinsic interest value, indicating that a semester’s transition can effectively help students develop greater intrinsic interest in senior high school grammar learning. This improvement is noteworthy because a strong intrinsic interest value is positively related to students’ self-concept of ability, which can contribute to their academic achievement (Denissen et al., 2007; Durik et al., 2006).

However, the qualitative data revealed a more complex picture. Despite the overall increase in this dimension, the low achievers, like Allen, expressed a negative attitude towards English learning. This may be explained by the influence of academic achievement on intrinsic interest, as previous research has suggested that low achievers express less interest than higher-achieving peers (Köller et al., 2001). Therefore, one semester of grammar instruction may not be sufficient for low-achieving students to develop a strong intrinsic interest, particularly during the transitional phase when students face great cognitive and emotional adjustments.

Similarly, the increase in extrinsic utility value, which reflects the perceived external benefits of grammar learning, implies that students’ awareness of the importance of grammar for their academic success has improved. The increase is important as utility value had a strong predictive effect on students’ educational aspirations (Guo et al., 2015). This suggests that recognizing the long-term usefulness of grammar learning is important in shaping their educational goals and sustained engagement.

The increase in students’ expectancies for success demonstrates that students gained greater confidence in their capacity to succeed in grammar learning tasks. The result is consistent with prior research on self-efficacy (Bandura, 1997), a closely related construct. In this study, the development of grammar-related confidence may reflect students’ adjustment to new instructional methods. However, qualitative findings suggest this growth may not have been linear. Students initially struggled with the shift from practice-based to concept-based instruction, which challenged their existing learning strategies. As teacher support increased, students gradually gained their belief in their ability to succeed.

However, the perceived attainment value did not change significantly over the course of the semester. This shows that students’ perception of the importance of achieving success in English grammar learning remained relatively stable during the transition period. One possible explanation lies in the nature of the construct. Attainment value is often associated with one’s identity (Tang et al., 2022; Wigfield et al., 2009), which tends to develop over a longer period. Therefore, one semester may not be sufficient to make noticeable changes in this dimension. Another possible explanation is that the perceived attainment value was already relatively high at T1 (Median = 3.33), which implies that students already considered English grammar learning highly important when entering senior high school. This may be due to the value placed on English proficiency in China, which is considered important at both the personal and societal levels (You and Dörnyei, 2016). Such high initial scores may have led to a ceiling effect, limiting the potential for detectable increases. This echoes the qualitative finding that attainment value remained a meaningful motivator, as students like Allen expressed a strong link between academic success and social image.

Additionally, the perceived task difficulty from the beginning to the end of the semester did not change significantly, although a slight decrease was observed in the upper quartile. This quantitative finding echoed the qualitative result that the perceived increase in grammar learning difficulty, due to the shift from junior to senior high school, could hinder students’ motivational adaptation during transition. In other words, students started the semester with an already high level of perceived difficulty, and a semester of learning failed to lower the perceived task difficulty. This may be attributed to the increase in content complexity of English grammar content from junior to senior high school. As a result, even though effective instructional support, such as explicit conceptual explanations, may have helped students cope with the learning challenges, they failed to make a significant reduction in perceived task difficulty.

According to the interview data, another unexpected phenomenon was found. Students who actually performed well in English, like Anna, still felt that they had not fully adapted to grammar learning. This finding demonstrates a possible mismatch between students’ objective academic performance and their subjective sense of adaptation. Students like Anna had associated grammar learning with easy success during junior high school, forming a strong belief in their competence. However, the increased complexity of senior high school grammar may have challenged this belief, even if their academic performance remained strong, causing them to feel less confident and less adapted despite high achievement. The perception is consistent with Wigfield et al. (1997), who suggested that students tend to place higher value on tasks in which they feel competent. When that sense of competence is challenged, the task may come to feel less valuable and more psychologically demanding, so that even high achievers may question whether they have truly adapted.

These findings offer implications for teaching practice and motivational support during the transition to senior high school. The findings highlight the importance of teacher guidance in supporting students’ adaptation. As students with various levels of academic achievement adjust to the new learning demands at different paces, tailored teaching strategies, such as the scaffolding strategy and process-oriented feedback, are recommended during the transitional period.

For low-achieving students like Allen, the scaffolding strategy is recommended to help them gradually understand and apply grammar concepts in different contexts. For instance, teachers can provide detailed grammatical examples before asking students to practice themselves. Scaffolding can help students find it easier to master grammatical knowledge, reducing their perceived learning difficulty and contributing to a smoother transition. Such learning processes help students gain positive learning experiences, increasing their expectancies for success. As students’ learning confidence increases, teachers then gradually withdraw their support and encourage students to apply the concepts independently.

For high-achieving students such as Anna, process-oriented feedback is needed to reduce the gap between their subjectively low self-evaluations and their objectively high academic performance. Teachers can monitor these students’ daily assignments more closely and give specific positive feedback. The feedback can help the students develop a more accurate perception of their learning, decreasing their feelings of competence threat.

Beyond classroom practice, these findings also have implications for teacher education. Teacher training programs should help pre-service teachers develop a deeper understanding of adolescents’ psychological development, especially during the transitional periods. They also need to be equipped with a variety of teaching strategies so that they can flexibly apply instructional approaches to students with different needs.

While this study offers insights into students’ adaptation to English grammar learning during the transition period, several limitations must be acknowledged. First, the small sample size of 80 questionnaire participants limits the generalizability of the findings to a larger population. Having six interviewees also restricts the diversity of perspectives found in the qualitative data. Besides, all participants were from the same class and were taught by the same teacher, which may limit the applicability of the results to other schools or regions. This may also bring about common contextual influences among students. Additionally, the study did not examine whether there were differences in change patterns across subgroups (e.g., whether EVT constructs changed differently for high and low achieving students). Last but not least, the study only examines the adaptation over a single semester, meaning that the longer-term effects of the transition remain unknown. Future research should consider a larger, more diverse sample and a longer period of observation to have a more comprehensive understanding of these effects.

## 6 Conclusion

This study investigated students’ motivational adaptation to the increased difficulty of senior high school English grammar. The findings reveal that students’ intrinsic interest value, extrinsic utility value, and expectancies for success increased significantly over the semester, while attainment value and perceived task difficulty remained relatively stable. Qualitative data further identified four major factors influencing students’ adaptation, namely, students’ affective attitudes towards English grammar learning, perceived increase in grammar difficulty, instructional support from teachers, and perceived self-worth value.

Together, these results underscore the importance of strengthening students’ psychological resources, particularly their motivation and confidence, as a means of supporting successful academic adaptation in demanding educational transitions.

## Data Availability

The raw data supporting the conclusions of this article will be made available by the authors, without undue reservation.
