# Supplementary material for: Motivational adaptation in English grammar learning: a mixed-methods study of students during the transition to senior high school
Source: Front Psychol. 2026 Jan 7;16:1703576. doi: 10.3389/fpsyg.2025.1703576 (PMC12819770; doi:10.3389/fpsyg.2025.1703576)
Supplement: Supplementary file 1 [file Data_Sheet_1.pdf]

# Appendices

## Appendix A. Questionnaire

We are pleased to invite you to participate in a survey examining how first-year senior high school students adapt to learning English grammar. Please respond to each statement based on your current learning experiences. Your responses will be kept confidential, so choose the option that best reflects your perceptions. Your participation is greatly appreciated.

[Response scale: ○ Strongly Disagree○ Disagree ○ Agree ○ Strongly Agree]

1.I believe I can do well in future English grammar lessons.

2.Being good at senior high school English grammar is important to me.

3.I believe I can successfully learn new English grammar topics.

4.I need to put in a lot of efforts to learn senior high school English grammar well.

5.I am confident in learning senior high school English grammar well with the English learning ability and experience I accumulated in junior high school.

6.In my spare time, I enjoy studying English, especially English grammar.

7.I often use English in real life.

8.I am confident in learning senior high school English, especially in terms of grammar.

9.I think senior high school English grammar learning will impact my future development.

10.Doing well in English grammar matters to me more than most other subjects.

11.English is very helpful in my daily life.

12.I find English grammar difficult to learn.

13.Compared to other subjects, I prefer studying English.

14.I would feel disappointed if I did not do well in English grammar.

15.I think senior high school English learning is enjoyable.

16.I think senior high school English grammar learning is enjoyable.

17.In real life, English is needed everywhere.

18.I can appreciate the beauty of English as a language.

19.It usually takes me a long time to grasp new grammar rules.

intrinsic interest value: 6, 13, 15,16, 18; extrinsic utility value: 7, 9, 11, 17; attainment value: 2, 10,14; expectancies for success: 1, 3, 5, 8; perceived task difficulty: 4, 12, 19

**Appendix B. Interview Guide for the Senior High School English Teacher**

1. In your grammar teaching, especially during the transition from junior high to senior high, have you encountered any common difficulties among students? Do you think these issues are related to the transition? How do you usually address them?

2. Are there any successful cases or experiences you can share that helped students overcome difficulties in learning grammar?

3. How do you design your English courses to ensure a smooth progression in students' grammar learning, especially for the transition between junior high and senior high?

4. Do you have specific teaching strategies that aim to promote students' gradual understanding and application of English grammar?

5. What specific teaching methods or activities do you use when teaching grammar to help students better comprehend and master it?

6. Are there any teaching resources you find effective for supporting students' transition in grammar learning between junior high and senior high?

7. As a senior high English teacher, are you familiar with junior high English instruction, such as textbooks, curriculum standards, exam formats, or teaching models?

8. Have you observed differences in students' needs regarding grammar learning in junior high versus senior high? Could you give some specific examples?

9. What differences do you think exist between English teaching in junior high and senior high? What is the biggest difference? Specifically regarding grammar teaching, what is the biggest difference?

10. Do you think transitional instruction in grammar is necessary for students entering Grade 10? What approaches can be used to facilitate the transition?

11. How do you assess students' grammar learning outcomes? Are there specific assessment tools or methods you use to ensure their mastery?

12. Are there any resources you would recommend to help other teachers better implement grammar teaching during the transitional period?

**Appendix C. Interview Guide for the Senior High School Freshmen**

1. Since entering senior high school, what new experiences or changes have you noticed in your English learning, especially grammar learning?

2. After one semester of study, do you feel you have adapted to English learning in senior high school, especially grammar learning? You may answer from perspectives such as emotions and attitude, learning strategies, and how well you accept the textbook and teaching methods.

3. Concerning the transition between junior and senior high school English grammar, do you feel you have encountered any difficulties? Why or why not? Have you overcome these difficulties?

4. Are there any specific grammar points or concepts that you find particularly challenging? If so, what do you think makes them difficult for you?

5. After one semester of study, what progress do you think you have made in grammar?

6. If you continue learning grammar at a similar pace as in your first semester, do you feel confident that you will have a good command of grammar by the time you reach Grade 12? Why or why not?

7. What measures have your English teacher taken, or what teaching tools have they used, in grammar teaching? Do you think these measures have helped you with the transition from junior to senior high school in terms of grammar learning?

8. What obvious differences do you find between grammar teaching in junior high school and in senior high school?

9. During this semester, have you participated in any grammar-related activities or projects, such as competitions? If yes, please share your experiences.

10. In your spare time, do you spend extra time learning English grammar? Why or why not? If so, how do you usually do it?

11. How important do you think learning English grammar is? In what ways do you think it may influence your future study and life?

**Appendix D. Summary of Themes, Sub-Codes, Definitions, and Quotes**

| Theme | Code | Definition | Quote |
|---|---|---|---|
| Affective attitudes towards English grammar learning | Dislike and avoidance of English learning | Strong negative emotions toward English learning and a clear tendency to avoid it | "I do not like English. To be honest, learning English is something I don't really want to do. I would rather practice two hours on math than memorize English words for half an hour." |
| | Emotional adjustment toward acceptance | The attempt to transform resistance into a more accepting attitude toward English learning | "But I am trying to turn English learning into something acceptable for me." |
| | Enthusiasm toward grammar learning | Strong motivation to master grammar despite its challenges | "I laid much more emphasis on English grammar learning. Although it is challenging, I am making every effort to be the 'grammar master'." |
| Perceived increase in grammar difficulty | Increased task complexity and learning demands | Greater analytical and integrative ability demands in senior high school grammar learning | "I learned English grammar mainly by doing exercises in junior high school. Although there was no systematic instruction, I still felt it was simple and I scored high marks. In senior high school, grammar is really important. Without a good command of grammar, it is hard to perform well in reading tasks." |

| | | |
|---|---|---|
| From drilling to conceptual understanding | A transition from mechanical drills to concept-based grammar learning | "My English teacher taught me grammar mainly by drilling in junior high school, without much focus on explaining the concepts behind it. Therefore, I just kept practicing different exercises. In senior high school the grammar concepts are clearly explained in class. Now, I think understanding the concepts and using them correctly in different contexts are more important." |
| Lack of conceptual preparation in junior high school | Increased senior high grammar learning difficulty caused by inadequate junior high grammar preparation | "One common problem most students encounter in senior high school is the lack of conceptual knowledge of English grammar. We need to take a lot of efforts to help them first understand these concepts. This step should have been done when they were in junior high school." |
| Perceived significant increase in difficulty | The perceived increase in English learning difficulty in senior high school | "In junior high school, English was really easy. I was quite satisfied with my senior high school entrance examination results. But in senior high school, it's a different story. The difficulty level has risen so much." |

| | | | |
|---|---|---|---|
| Instructional support from teachers | Peer learning through example display | The use of students' work as examples to facilitate grammar learning | "My teacher displays my classmates' homework in the form of photos, allowing us to learn from their problem-solving methods, since there are marks on the papers." |
| | Detailed conceptual explanation | Support for grammar learning by detailed explanations of grammatical concepts | "She also goes through each question in detail, helping us understand the underlying grammatical concepts." |
| | Active student participation in error analysis | Active explanation of grammatical knowledge to peers | "One effective method my English teacher uses is called the 'Error Case Report.' Each student selects a representative grammar error from their daily practice, prepares relevant materials, and explains it to the whole class." |
| | Learning benefits of reflective error analysis | Benefits of reflective error analysis for students' understanding of grammar rules | "This process not only deepens my understanding of the error itself but also helps me recognize similar issues in others' work." |
| Perceived self-worth value | Effort motivated by | Study motivation related to maintaining a | "I am the class monitor, and if I perform poorly in English, then it will be really embarrassing. |

| | | | |
|---|---|---|---|
| | self-worth concerns | positive peer image | Therefore, I need to work harder to catch up with the other classmates.” |
